# Soil Pollution and Remediation

**DOI:** 10.3390/ijerph15081657

**Published:** 2018-08-05

**Authors:** Jorge Paz-Ferreiro, Gabriel Gascó, Ana Méndez, Suzie M. Reichman

**Affiliations:** 1 School of Engineering, RMIT University, GP.O. Box 2476, Melbourne VIC 3001, Australia; suzie.reichman@rmit.edu.au; 2 Centre for Environmental Sustainability and Remediation, RMIT University, GP.O. Box 2476, Melbourne VIC 3001, Australia; 3 Departamento de Producción Agraria, E.T.S.I. Agronómica, Alimentaria y de Biosistemas, Universidad Politécnica de Madrid, Ciudad Universitaria, 28004 Madrid, Spain; gabriel.gasco@upm.es; 4 Departamento de Ingeniería Geológica y Minera, E.T.S.I. Minas y Energía, Universidad Politécnica de Madrid, C/Ríos Rosas No. 21, 28003 Madrid, Spain; anamaria.mendez@upm.es

Industrialized economies and developing countries are affected by soil pollution originating from mining, industrial activities, improper disposal of waste, and mechanized agriculture. Soil pollution could lead to impacts on crop productivity and human health. Investigating the sources, fate and occurrence of soil pollution, and the risks posed to human health has thus been an important area of research. 

An increasing amount of research has been devoted to finding innovative and sustainable solutions for the remediation of contaminated land. The multiple challenges associated with the remediation of polluted soils have been overcome by the use of soil amendments, thermal desorption, soil washing, electrokinetic remediation, and bioremediation.

This Special Issue collects research papers aimed at a wide range of soil remediation topics, presenting an integrated view of the trends in solving the problems associated with the obtaining successful soil remediation. This special issue contains eleven articles that have been selected as emerging studies dealing with the above-mentioned topics. 

Two of the articles presented in this special issue were directly devoted to assisted phytoremediation. Phytoremediation has been increasingly used in recent years as it has significant co-benefits, including providing a plant cover to the soil which contributes to reduced erosion. The success of phytoremediation approaches depends ultimately on plant growth and the concentration of the element of interest absorbed by the plant. Thus, Radziemska et al. [1] used different materials for assisted phytostabilization, including diatomites, halloysite, and chalcedonite. They found the last two to be more effective when combined with *Brassica napus*. Huang et al. [2] pyrolyzed organic matter, a technique which can contribute to reduce waste streams while producing a material with better characteristics for soil addition called biochar. Biochar has been used as a soil amendment due to its ability to immobilise heavy metals, including Zn [3] and Cd [4], while contributing to carbon sequestration [5]. Huang et al. [2] produced three biochars with different characteristics which were then trialled for assisted phytostabilization using *Cassia alata*, of a mine tailing contaminated with several heavy metals. Their biochar improved plant growth and generally enhanced the root uptake of several elements. However, the risk of As release was observed at higher biochar doses.

The addition of innovative materials to the soil was also a focus of Jiang et al.’s study [6]. A nanometallic aluminium/calcium oxide dispersion mixture was shown to be highly efficient for the dechlorination of hexachlorobenzene. The material prepared by these authors has the potential to result in a more cost-effective remediation than the more commonly utilised Ca/CaO dispersion mixtures.

Structural differences in pollution patterns were a focus of several articles in this special issue. Skala et al. [7] studied the contribution of organochlorine pesticides, polycyclic aromatic hydrocarbons, and polychlorinated biphenyls to human health risks in floodplains. Health risk assessment was also a focus of the study by Retamel-Salgado et al. [8]. In this case, the health risk posed by the consumption of maize growing in three soils with contrasting characteristics polluted with cadmium was evaluated. 

Wang and Zhang [9] studied the influence of soil properties, vegetation, and road age on heavy metal concentrations in roadside soils in Hangzhou. Hu et al [10] and Jia et al. [11] studied the spatial variability of heavy metals in soils using geostatistics. Zhou et al. [12] studied the variability of chromate in a topsoil and its corresponding subsoil concentrations.

Yang et al. [13] used composted sewage sludge as an additive to an urban garden soil. Total concentrations, nonresidual fractions, and bioaccessibility of Cr, Cu, Pb, and Zn increased after addition of composted sewage sludge to the soil. 

Zhu et al. [14] studied Ni toxicity to barley and correlated it with a single EDTA extraction and three sequential extractions with EDTA. They found that multiple extractions improved the prediction of the toxicity models. 

This body of work presented in this special issue shows the potential for assessing and remediating polluted soils. The work showcased to stakeholders how innovative systems can be integrated into traditional soil remediation
